# Photoluminescence Study of Undoped and Eu-Doped Alkali-Niobate Aluminosilicate Glasses and Glass-Ceramics

**DOI:** 10.3390/ma17102283

**Published:** 2024-05-11

**Authors:** Maria Rita Cicconi, Hongyi Deng, Takahito Otsuka, Aadhitya Telakula Mahesh, Neamul Hayet Khansur, Tomokatsu Hayakawa, Dominique de Ligny

**Affiliations:** 1 Department of Materials Science and Engineering, Institut für Glas und Keramik, Friedrich-Alexander-Universität Erlangen-Nürnberg, 91058 Erlangen, Germany; hongyi.deng@fau.de (H.D.); aadhityatm@gmail.com (A.T.M.); neamul.khansur@fau.de (N.H.K.); dominique.de.ligny@fau.de (D.d.L.); 2 Department of Life Science and Applied Chemistry, Nagoya Institute of Technology, Nagoya 466-8555, Japan; t.otsuka.098@nitech.jp (T.O.); hayakawa.tomokatsu@nitech.ac.jp (T.H.)

**Keywords:** self-activated emitting phosphors, JO intensity parameters, photoluminescence lifetime, KNN, perovskite glass-ceramics

## Abstract

In this study, the photoluminescence (PL) behavior of two aluminosilicate glass series containing alkali-niobates ranging from 0.4 to 20 mol% was investigated. The glasses exhibit an intense visible emission centered at ~18,400 cm^−1^ for the peralkaline series and at higher energies (~19,300 cm^−1^) for the metaluminous glasses. However, the photoluminescence emission intensity varies significantly with the niobate content and the bulk chemistry. PL and fluorescence lifetime measurements indicate that the broad emission bands result from the overlap of different niobate populations, whose distribution changes with niobate content. The distinct PL behavior in the two glass series was related to the structural evolution of the niobate units upon niobium addition. An enhancement of the visible emission was observed for a higher fraction of distorted [NbO_6_] units. Eu-doping was carried out as a structural probe of the glass network, and also to determine if these glasses could be used as potential rare earth element (REE) activators. The crystal field strength around Eu ions is strongly dependent on the bulk chemistry and the niobate content. Furthermore, the peralkaline series showed energy transfer from the host [NbO_6_] to Eu^3+^, confirming the feasibility of exploring niobate glasses and glass-ceramics as lanthanide ion-activated luminescent materials. In addition, glass-ceramics (GCs) containing alkali-niobate phases with a perovskite-like structure were developed and studied to verify the optical performance of these materials. It was verified that the bulk chemistry influences crystallization behavior, and also the photoluminescence response. The transparent GC from the metaluminous series exhibits a quenching of the Eu^3+^ emission, whereas an enhanced emission intensity is observed for the peralkaline GC. The latter shows a strong excitation-dependent PL emission, suggesting energy transfer and migration of electronic excitation from one Eu population to another. Additionally, Eu^3+^ emissions arising from the D15 and D25 excited states were observed, highlighting the low phonon energy achievable in niobo-aluminosilicate hosts.

## 1. Introduction

Niobate-bearing materials have many interesting applications, ranging from piezoelectric/pyroelectric properties, energy storage (dielectric capacitors), electro-optical coupling, photorefractive applications, etc. [1,2,3]. The most studied and known material is the LiNbO_3_ phase, which possesses a combination of distinctive characteristics, such as large electro-optical and electro-mechanical coupling, and photorefractive and non-linear optical properties [2,3]. Other niobate-based ceramics are considered crucial in developing sustainable, environmentally friendly ferroelectric systems. In particular (K,Na)NbO_3_-based ceramics are the most promising candidates to replace the toxic lead titanate-zirconate and lead-niobate ferroelectrics [4,5]. KNbO_3_ and NaNbO_3_ form a continuous solid solution, and regardless of the K/Na molar ratio, all phases possess a perovskite structure. However, they have distinct symmetries due to the different octahedral tilting [5], and the piezoelectric properties are maximized in the proximity of the equimolar K/Na: the (K_0.5_Na_0.5_)NbO_3_ (hereafter KNN) composition. Unfortunately, both the structure and properties of (K, Na)NbO_3_-based ceramics are quite sensitive to processing conditions, thus reducing reproducibility and reliability [4,5]. The development of glass-ceramics containing these functional crystals would improve large-scale manufacturing and allow a combination of properties for, i.e., applications in optics and innovative energy-related technologies (e.g., second harmonic generation, down- and up-conversion, pyroelectricity, piezoelectricity, high energy-storage dielectric, etc.).

Niobium, a versatile element in glasses, has an intermediated structural behavior and provides high linear and non-linear refractive indices. Indeed, in oxide glasses, the addition of *d*^0^ elements (Nb^5+^, Ti^4+^, W^6+^) allows a substantial increase in the non-linear optical response (~10 times greater than silica), and glasses containing Nb^5+^ ions have attracted much attention for their enhanced third-order nonlinearity [6]. Therefore, the introduction of niobium in glasses has been extensively studied in silicate, borate, germanate, and phosphate systems because they are optimal candidates as non-linear photonic materials; unfortunately, there are few systematic studies of non-binary glass systems in the literature [7,8,9,10]. To fill this gap, a comprehensive study of niobium incorporation in alkali aluminosilicate glasses with different alkali/aluminum (polymerization) ratios has recently been carried out [11]. It was observed that the Nb structural environment evolves differently depending on the bulk chemistry and that the existence of distinct Nb populations has a significant impact on property and crystallization behavior.

In recent years, numerous studies have focused on the development of glasses and ferroelectric glass-ceramics (GCs) for a variety of applications, ranging from electro-optic to high-voltage capacitors [12,13,14]. Yet, there have been limited investigations into the photoluminescence behavior. Indeed, despite a substantial amount of published research, there is a significant scarcity of information regarding the luminescence properties of niobate units in glasses and glass-ceramics. The fact that the earliest (claimed) report of this phenomenon in glasses is from 1998 [15] is quite surprising. It is even more astonishing considering that Blasse and colleagues have detailed the impact of the niobate crystal structure on the absorption edge and photoluminescence emission properties since the 1960s [16,17,18]. These research works indicate that an intense and tunable visible emission is observed under UV irradiation, depending on the Nb^5+^ polyhedral distortion and connectivity (isolated NbO_6_ units, edge/face sharing, corner shared NbO_6_). The proposed emission mechanism involves the transfer of an electron from ligand to metal (LM charge transfer); therefore, Nb^5+^ ions accept excited electrons from *2p* orbitals of O^2−^. Examples of compounds with the same emission mechanism are YTaO_4_ and CaWO_4_, self-activated blue-emitting phosphors, and well-known host lattices for efficient luminescence materials (lanthanide ion-activated luminescent materials) [19,20]. As mentioned, in glasses, Nb^5+^ photoluminescence studies are scarce. In a soda–lime–silica glass, it was recently proposed that adding closed-shell transition metal ions (*d^0^* configuration, such as Nb^5+^) could find applications in cover glasses for photovoltaic devices, to enhance UV protection of polymeric PV module components [21].

In this study, it is investigated if the UV down-shifting to visible photons provided by Nb^5+^ ions could make them also good candidates as lanthanide ion-activated luminescent materials (in short, activators). This could find a broad range of applications in sensors, solid-state lasers, up-conversion luminescence, and so on, similar to the well-known YVO_4_-Eu^2+^. Still, the limited knowledge of the emission in different glass matrices and the derived glass-ceramics hamper any further improvement, and further studies are needed. Therefore, in the present study, two glass series having different polymerizations and containing alkali niobates (KNN) from 0.4 mol% up to 20 mol% were investigated with the aim to (i) provide the photoluminescence behavior of niobo-aluminosilicate glasses, (ii) verify if these glasses could be used as potential lanthanide ion-activated luminescent materials, and iii) study the optical performance of the glass-ceramics.

## 2. Experimental Methods

Details of the glass synthesis, bulk chemistries, and physical and thermal properties, along with the study of the glass structure, are reported in [11].

Pristine glasses have compositions corresponding to the ternary SiO_2_–Na_2_O–Al_2_O_3_ system, with constant SiO_2_ molar content (~66.6 mol%) and Na_2_O/Al_2_O_3_ molar ratios of 2.3 and 1, respectively corresponding to a composition in the peralkaline region (Na_2_O > Al_2_O_3_, sample label NA66.10), and a polymerized glass at the metaluminous joint (Na_2_O = Al_2_O_3_, label NA66.17). Portions of the pristine glass were finely ground, doped with commercially available K_0.5_Na_0.5_NbO_3_ (KNN) from 0.4 up to 20 mol%, then remelted in air with a dwell time of 1 h at 1500 °C and 1650 °C, respectively for NA66.10 and NA66.17, corresponding to nearly isoviscous conditions [11]. The Nb_2_O_5_ content of the glasses studied here is reported in Table 1 (compositions from ICP analysis; from [11]). Relaxed samples were ground down to a thickness of ~0.5 mm and polished to optical quality. Three samples having different KNN contents from each glass series (3 mol% KNN, 5 mol% KNN, and 10 mol% KNN) were finely ground and doped with 0.18 mol% Eu_2_O_3_. The homogenized powder was remelted at the same conditions reported above for one hour.

Subsequent thermal treatment of the glass specimens was carried out to produce glass-ceramics (GCs). Polished glass specimens were heated up to the exothermic event with a 10 K/min heating rate, and kept at the crystallization temperature for 2 to 4 h.

X-ray powder diffraction patterns were collected for all crystallized samples by an X-ray diffractometer (Bruker D8 ADVANCE Eco, Brucker AXS GmbH, Karlsruhe, Germany), equipped with a Cu source. Diffraction patterns were recorded in the 2θ range of 15–80° (step size ~0.02°, 1 s acquisition time) using a position-sensitive detector LYNXEYE-XE. The TOPAS (version 5, Brucker AXS GmbH, Karlsruhe, Germany) was used to model the diffraction. The Fundamental Parameter Approach (FPA) was used to model the instrument peak profile.

Optical absorption spectra were measured using a UV–Vis–NIR spectrometer (Lambda 950, PerkinElmer, Waltham, MA, USA) in the ~200 to 850 nm range. The absorption coefficient (α) was derived by considering the samples’ thickness [11]. Analyses of optical emission and excitation behavior were performed with a spectrofluorometer (Fluorolog3, Horiba Jobin Yvon, Horiba Europe GmbH, Oberursel, Germany) equipped with a double grating monochromator (Czerny-Turner) in both excitation and emission channels, using a 450 W Xe-lamp as the excitation source. All spectra were corrected for the PMT sensitivity and the Xe lamp’s output power. All measurements were carried out on polished glasses with dimensions of ~1.0 cm^2^ and ~0.5 mm thickness. Photoluminescence decay curves were obtained by using a pulsed 70 W pulsed Xe lamp as the excitation source, and data analysis was carried out by using the fluorescence decay analysis software DAS6 (Horiba Scientific, Horiba Europe GmbH, Oberursel, Germany).

## 3. Results and Discussion

### 3.1. Absorption and Photoluminescence Study of Alkali-Niobate Aluminosilicate Glasses

**Figure 1:**
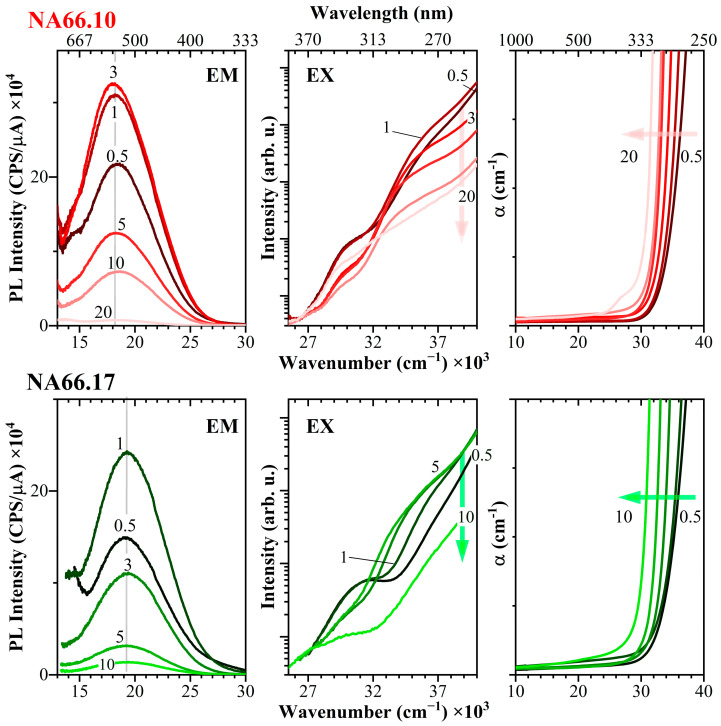
Photoluminescence emission (EM), excitation (EX), and optical absorption curves, collected at room temperature, for the two glass series having increasing KNN contents (exit and entrance slits = 3 nm). The vertical grey solid line highlights the blue shift in the emission signals for NA66.17 compositions compared to NA66.10 glasses. λ_ex_ = maximum of the shoulder in the excitation curve (270–285 nm); λ_em_ = maximum of the emission curve (515–555 nm).

Figure 1 reports the photoluminescence emission (EM), excitation (EX), and Optical Absorption (OA) curves, collected at room temperature, for the two glass series having increasing KNN contents. The frequency position of the absorption edge and details of the emission and excitation bands are reported in Table 1, along with the Stoke Shifts.

The absorption spectra reported in Figure 1 show very good transparency in the visible and near-infrared regions for all glasses. Additionally, a general trend in the two glass series can be observed, with the shift toward lower energies of the absorption edge by increasing KNN content and the occurrence of an absorption band around 27,200 cm^−1^ at the highest KNN content (see [11] for details). All KNN-doped glasses display intense visible emission when excited under a UV light, even at room temperature. The optical absorption and photoluminescence EM are usually associated with charge transfer transitions of electrons from O^2−^ (*2p* orbitals) to the empty *d* orbitals of the Nb^5+^ ions. The photoluminescence EM is very broad (FWHM > 5000 cm^−1^) and centered at ~18,400 cm^−1^ for the peralkaline series and at higher energies (~19,300 cm^−1^) in the metaluminous one. On the one hand, the position of the maximum barely changes for KNN contents from 0.5 to 20 mol% and only small variations in the broadness can be detected. On the other hand, the emission intensity drastically changes, reaching a maximum at 3 mol% KNN in NA66.10 and already at 1 mol% KNN in the polymerized glass NA66.17. The photoluminescence excitation signals collected at the maximum of the emission (EX, Figure 1) show the presence of two contributions (at ~29,400 cm^−1^ and 34,000–36,000 cm^−1^) whose relative intensities change depending on the KNN content. In particular, the high-energy one moves toward lower energies and becomes weaker at higher niobate contents.

**Figure 2:**
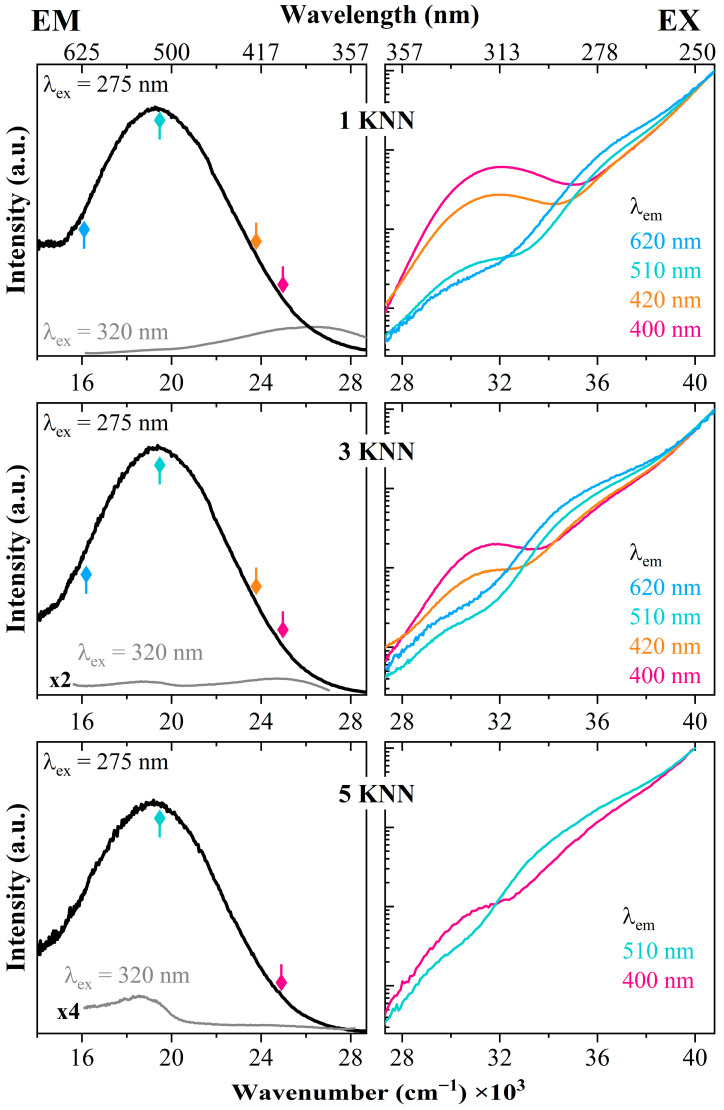
Room temperature photoluminescence emission (EM) and excitation (EX) spectra for NA66.17 glasses having increasing KNN contents (exit and entrance slits = 3 nm). The broad emission curves derive from the overlap of at least two populations. The two corresponding excitation centers peak around 36,200 cm^−1^ and 30,400 cm^−1^. The latter contribution strongly decreases by increasing KNN content. λ_em_ = 420 and 620 nm for sample 5KNN are not reported for the sake of clarity as the signals overlap.

Because both emission and excitation show broad bands, we collected the photoluminescence emission by using different slit widths (3 nm, 2 nm, and 1 nm) and the excitation spectra at different positions along the emission band to determine the presence of different convoluted centers. Figure S1 shows the emission spectra of samples in the NA66.10 series containing 1 and 3 mol% KNN collected with different slit widths (λ_ex_ = 275 nm). It is clear that the whole emission becomes narrower by decreasing the slit size, and, in particular, the shoulder at lower frequencies (~16,400 cm^−1^) becomes less important, resulting in an apparent shift toward higher energies of the emission. To understand if the different centers could be resolved, excitation spectra were collected at different positions along the emission band (400 nm, 420 nm, 510 nm, and 620 nm) for glasses having three different KNN contents (Figure 2). Depending on the selected position along the emission band, the excitation shows a clear change. Figure 2 shows the different relative intensities of the two excitation bands depending on the emission wavelength selected (both lamp-corrected and -uncorrected signals are shown in Figure S2). On the high-energy side of the EM band (λ_em_ = 400–420 nm), the strongest excitation center peaks around 30,000 cm^−1^, whereas on the maximum and on the low-energy side of the EM band (λ_em_ = 510 and 620 nm), the strongest excitation center peaks around 36,000 cm^−1^. Furthermore, by increasing KNN content, the excitation center at lower frequencies systematically decreases. These results, therefore, suggest that the emission curves derive from the overlap of at least two populations, one of which (the one at 30,000 cm^−1^) is more prominent at lower niobate contents.

**Figure 3:**
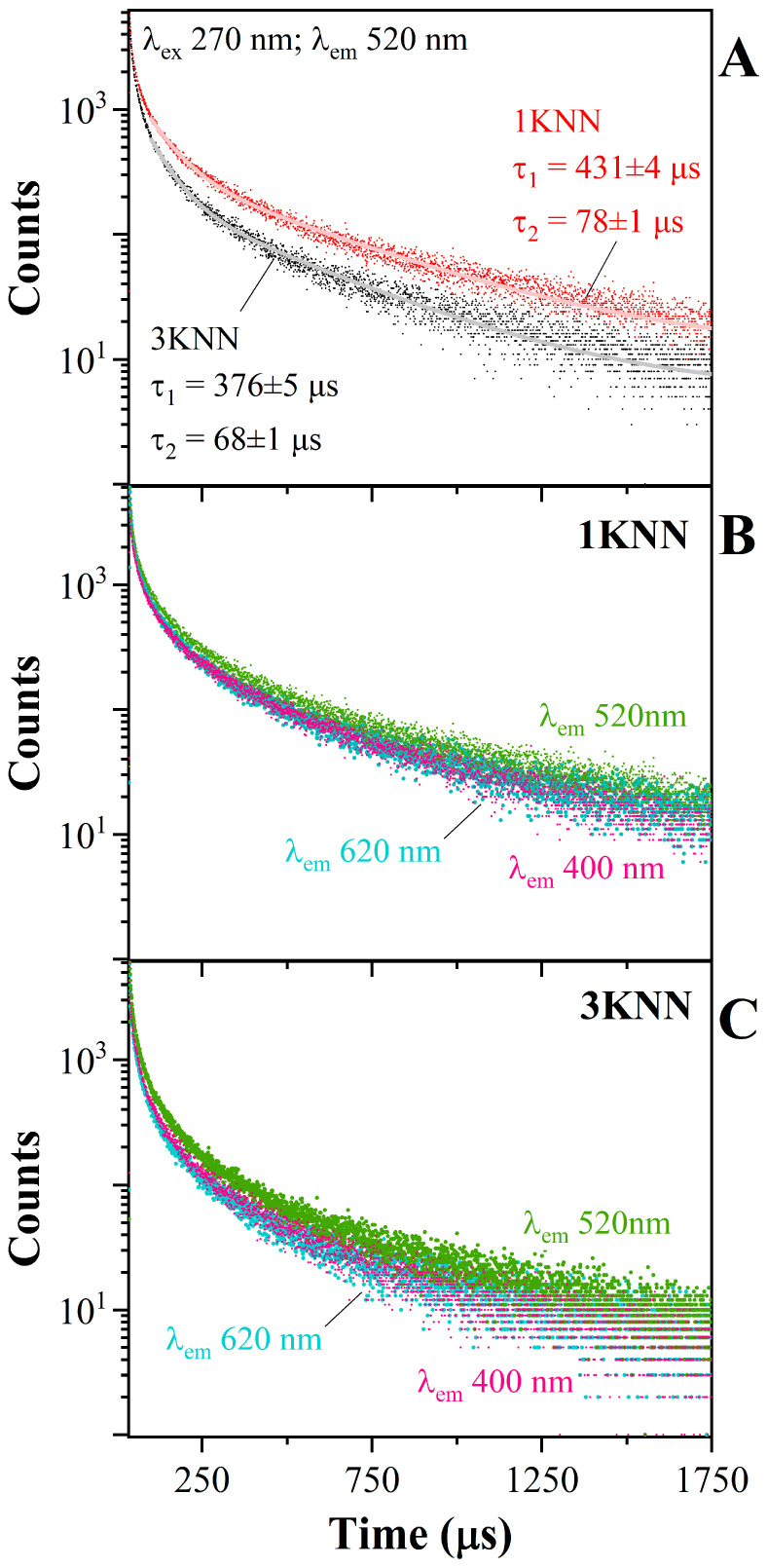
(**A**) Fluorescence decay curves of samples NA66.10_1KNN and _3KNN (under 270 nm excitation). The curves show a non-mono-exponential decay, comprising both fast and long decay components. (**B**,**C**) The decay curves collected at different emission wavelengths (on the high- and low-energy sides of the EM maximum for samples having 1 and 3KNN). There is a variation in lifetime, with a decrease in the long decay component supporting the existence of multiple niobate subpopulations.

Fluorescence lifetime measurements were carried out on the samples with the strongest emission (NA66.10_1KNN and _3KNN) at various positions along the emission band (under 270 nm excitation) (Figure 3). All curves show a non-mono-exponential decay, comprising both fast and long decay components, supporting the existence of multiple subpopulations. A double exponential function appropriately describes the decay curves collected close to the maximum of the emission band (λ_em_ = 520 nm), resulting in a lifetime of 431 ± 4 μs and 78 ± 1 μs, respectively, for long and fast decay of the 1KNN sample. By increasing KNN, there is a clear decrease in the lifetime: 376 ± 5 μs and 68 ± 1 μs, respectively, for the long and fast decay components, confirming that the connectivity of the niobate entities changes by increasing the KNN content.

Additionally, there is a variation in the decay components across different emission wavelengths. Indeed, the decay curves show a shorter lifetime when using emission wavelengths on the high- and low-energy sides of the EM maximum (Figure 3B,C), reinforcing the evidence that the broad emission bands derive from the overlap of different niobate populations, which distribution changes depending on the Nb_2_O_5_ content.

**Figure 4:**
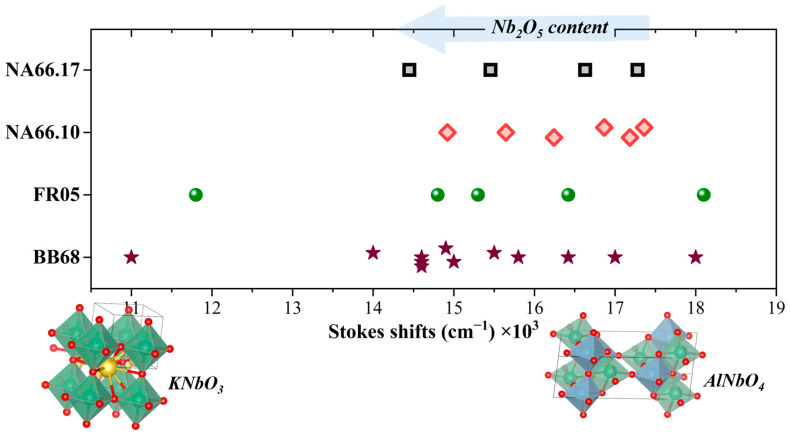
The Stokes shifts (SSs) (cm^−1^) of the two glass series (values reported in Table 1) are compared to Nb-crystalline compounds (BB68; [18]) and to La-borate glasses (FR05, [25]). In crystalline compounds, by decreasing the site distortion and, for example, moving from the distorted edge-shared polyhedral structure of AlNbO_4_ to a more regular corner-shared octahedral (KnbO_3_), the SS decreases from ~17,000 to ~11,000 cm^−1^. In the NA66.y glasses, by increasing the niobate content, there is a systematic decrease in the SS, in agreement with the structural data and, in turn, to the progression to less distorted corner-shared [NbO_6_] units.

In order to try to rationalize the photoluminescence behavior of the niobate-bearing glasses and, in turn, the relationship with the connectivity and distortion of the niobate entities, it is advisable to explore studies carried out on non-amorphous samples. Crystalline materials have been the subject of extensive investigation regarding the photoluminescence properties of niobates, particularly since the early studies by Blasse and coauthors [18,22,23]. These authors have reported that the local environment of Nb (coordination, polyhedral connectivity, and distortion) has a remarkable effect on both the position of the absorption edge and PL emission, with the latter ranging from 19,000 to 27,000 cm^−1^, depending on the crystal structure. Indeed, since the mechanism responsible for the PL is attributed to the charge transfer transition of electrons from O^2−^ to Nb^5+^ ions, it is very sensitive to variations in the emitter surroundings. For instance, when excited under UV light, Nb compounds with a columbite structure display an intense emission at room temperature; in contrast, crystals having a more regular corner-shared structure show a quenching of the photoluminescence emission. From the studies of several crystalline materials, a general trend can be drawn: in the case of isolated units, edge- or face-shared units, the emission band has been reported to be very efficient with rather large Stokes shifts. On the other hand, in corner-shared units with greater symmetry, the quenched emission is linked to a shift towards lower frequencies of both the emission and absorption edges, along with a notable reduction in the Stokes shifts [24,25,26,27]. In La_2_O_3_-B_2_O_3_ glasses having different Nb_2_O_5_ contents [15,25], the presence of a broad emission band at ~490 nm (~20,400 cm^−1^) upon UV excitation (280 nm), which shifted toward lower frequencies for increasing Nb_2_O_5_ contents, was observed. The variation in the Stokes shifts, depending on the crystalline structure or depending on the Nb content in La-borate glasses, is reported in Figure 4.

The two glass series studied here shows an energy decrease in the absorption edge with increasing Nb_2_O_5_ content, as shown in Figure 1. At the same time, there is a strong drop in the photoluminescence intensity but rather limited changes in the frequency position of the EM band. Nevertheless, it is clear that the broad emission derives from the overlap of different centers. To compare these results to prior literature, we used the Stokes shifts (SSs) (Figure 4). As reported above, in crystalline compounds, depending on the polyhedral connectivity and distortion, the Stokes shift varies strongly. For example, AlNbO_4_ has highly distorted [NbO_6_] units with four edges shared, and has a broad emission centered around 23,000 cm^−1^ and SS ~17,000 cm^−1^ [18]. Instead, perovskite-like compounds ANbO_3_ (A = alkali) have a very weak emission at room temperature, centered around 19,000 cm^−1^, and very small Stokes shifts (~11,000 cm^−1^) (data for KNbO_3_ from [27]).

In the glass samples studied here, there is a systematic decrease in the Stokes shifts with increasing niobate content (Figure 4, Table 1). The structural investigation of these niobo-aluminosilicate glasses (NMR [28] and Raman data [11]) highlighted an evolution of the Nb local environment with increasing KNN contents, and, in particular, a progression from a heterogeneous environment with isolated octahedral units of varying distortion degrees, to Nb–O–Nb(Si) linkages, up to the formation of corner-shared octahedral clusters. This progression was similarly observed in other amorphous systems, including sodium borophosphate glasses [7] and niobium alkali-silicates [29]. Additionally, the optical absorption data of the aluminosilicate glasses showed that the band tail energy (Urbach energy) decreases when the KNN content increases [11], indicating that these glasses adopt indeed a less distorted structure. Therefore, the decrease in the Stokes shifts can be considered a fingerprint for a more ordered local environment in both niobate-crystalline materials and glasses. As a final remark, it is recommended that an alkali-rich glass matrix be used for enhanced photoluminescence purposes instead of a fully polymerized one. Indeed, the exceeding amount of, e.g., Na, contributes to the charge balance of the sparse Nb units, and a higher fraction of distorted [NbO_6_] entities, which provide stronger EM intensity. These glasses also exhibit an enhanced Raman scattering and a lower phonon energy than silicate and phosphate glasses [11].

### 3.2. Photoluminescence of Eu-Doped Alkali-Niobate Aluminosilicate Glasses

#### 3.2.1. Eu-Doped Pristine Glasses

**Figure 5:**
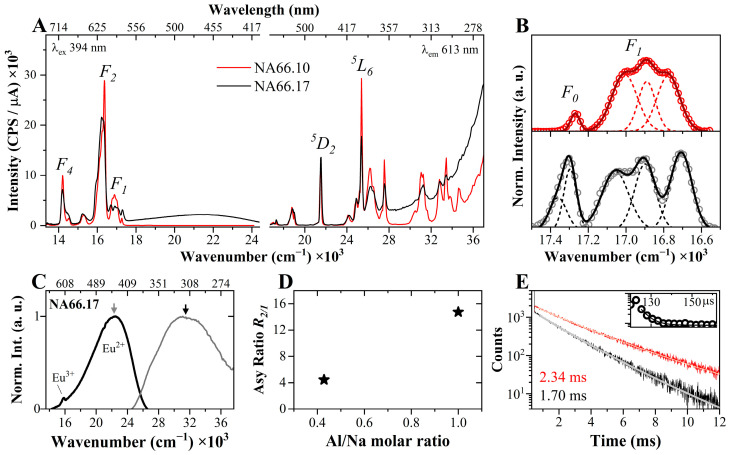
Photoluminescence results of the Eu-doped NA66.10 and NA66.17 pristine glasses (exit and entrance slits = 1 nm): (**A**) Eu^3+^ transitions in the emission and excitation spectra. (**B**) Deconvolution of the D0 →5F07 and →F17 transitions using Gaussian functions; in particular, the $F_{0}$ transition of NA66.17 glass shows the presence of two components (populations). (**C**) Excitation and emission spectra collected at the respective maxima (vertical arrows at 320 and 440 nm) associated with Eu^2+^ fluorescence in NA66.17. (**D**) The asymmetricity ratio $R_{2/1}$ confirms the strong changes in the Eu^3+^ local symmetry depending on glass polymerization. (**E**) Fluorescence decay curves at 613 nm (under 394 nm excitation) for the two Eu-doped pristine glasses. The curves show a mono-exponential decay, but NA66.17 has additionally a very short-rise component (inset in **E**).

To gain more insight into the structure of the niobate species and to verify if the niobate-related photoluminescence could be exploited as a rare earth element (REE) activator, the two series were doped with 0.18 mol% Eu_2_O_3_. First of all, in the pristine glasses, it is expected that they have a different Eu photoluminescence behavior because of the different glass polymerizations, which induces variations in the Eu^3+^ surrounding it. In fact, the photoluminescence excitation (EX) and emission (EM) spectra reported in Figure 5A show large differences. The excitation, collected under λ_em_ = 613 nm, consists of several sharp peaks assigned to the *f–f* transitions from the ground state (F07) of the Eu^3+^ ions [30]. The most intense transition is centered at ~25,380 cm^−1^ and corresponds to the F0 →7L65 excited state. The other prominent bands are located at ~27,550 cm^−1^ (F0 →7D45), ~21,550 cm^−1^ (F0 →7D25), and ~18,840 cm^−1^ (F0 →7D15). The photoluminescence EM spectra show the typical strong and sharp electronic transitions D0 →5FJ 7(J=0, 1, 2, 3, 4). The most intense band is the electric-dipole D0 →5F2 7 band, located at ~16,300 cm^−1^, which is hypersensitive, i.e., strongly affected by the site symmetry of the Eu^3+^ ions [30,31,32]. Both the EX and EM spectra of the glasses show noticeable variations in terms of bandwidth and relative intensities. In particular, in the EM spectra, the energy split of the Stark components is rather different, as well the broadness (and asymmetricity) of the F07 transition, which is very useful in the determination of the presence of different Eu populations [30,33]. To better visualize these variations, and to gain better insight into the presence of different Eu^3+^ populations and crystal field variations, the magnetic-dipole transition (D0 →5F17) and the *J*-mixing D0 →5F07 transitions have been deconvoluted (Figure 5B). Three Gaussian were used to deconvolute the $F_{1}$ transition in both glasses, but the stronger splitting in the polymerized NA66.17 sample is rather obvious. The energy difference between the components in the polymerized glass indicates the presence of a strong crystal field and low symmetry around Eu^3+^. Furthermore, two components are needed to analyze the $F_{0}$ band, indicating the presence of at least two main Eu^3+^ populations. Another uniqueness of the polymerized NA66.17 glass is the presence in the emission spectrum of a broad band located at higher energies (centered around 22,700 cm^−1^). This band is attributed to Eu^2+^ species (Figure 5C), and is not present in the peralkaline composition. Based on previous studies dedicated to the redox behavior of Eu in silicate glasses, the presence of reduced species in this polymerized sample is expected [34,35].

To evaluate the different Eu^3+^ local environments, the well-known asymmetricity ratio $R_{2/1}$ was calculated from the integrated areas of the frequency ranges associated with the $F_{2}$ and $F_{1}$ transitions (Figure 5D):

(1) $$R_{2/1}=\frac{\int_{16570}^{15711}I_{F_{2}}\left(\omega \right)d\omega }{\int_{17200}^{16570}I_{F_{1}}\left(\omega \right)d\omega }$$

According to the literature on glass and crystalline materials, high asymmetricity ratio values indicate a high degree of distortion in the Eu^3+^ local environment [31]; furthermore, increasing $R_{2/1}$ values may also indicate an increase in the Eu-O bonding covalency character [30,36]. In the two glass series, the $R_{2/1}$ is much higher in the metaluminous glass, confirming the strong influence of the glass polymerizations on the Eu^3+^ structural environment. Even if a difference was expected, the polymerized glass has an extremely high $R_{2/1}$ value (>13), whereas the peralkaline glass has a value consistent with previous studies, e.g., [37]. A possible explanation for such a high $R_{2/1}$, besides a local environment with very low symmetry, involves the (at least) two Eu^3+^ sub-populations present in the NA66.17 glass, which would cause additional transitions below the $F_{2}$, and in turn, a higher integrated area. The strong differences in the local surrounding of the Eu^3+^ ions depending on the bulk chemistry are also confirmed by the lifetime measurements (Figure 5E), where the decay in the peralkaline glass is much longer (2.34 ± 0.01 ms) compared to that in the NA66.17 glass (1.70 ± 0.03 ms). The latter also has a very short-rise component (see inset in Figure 5E), that is exponential, with a lifetime of a few μs.

#### 3.2.2. Eu-Doped Niobate Glasses

**Figure 6:**
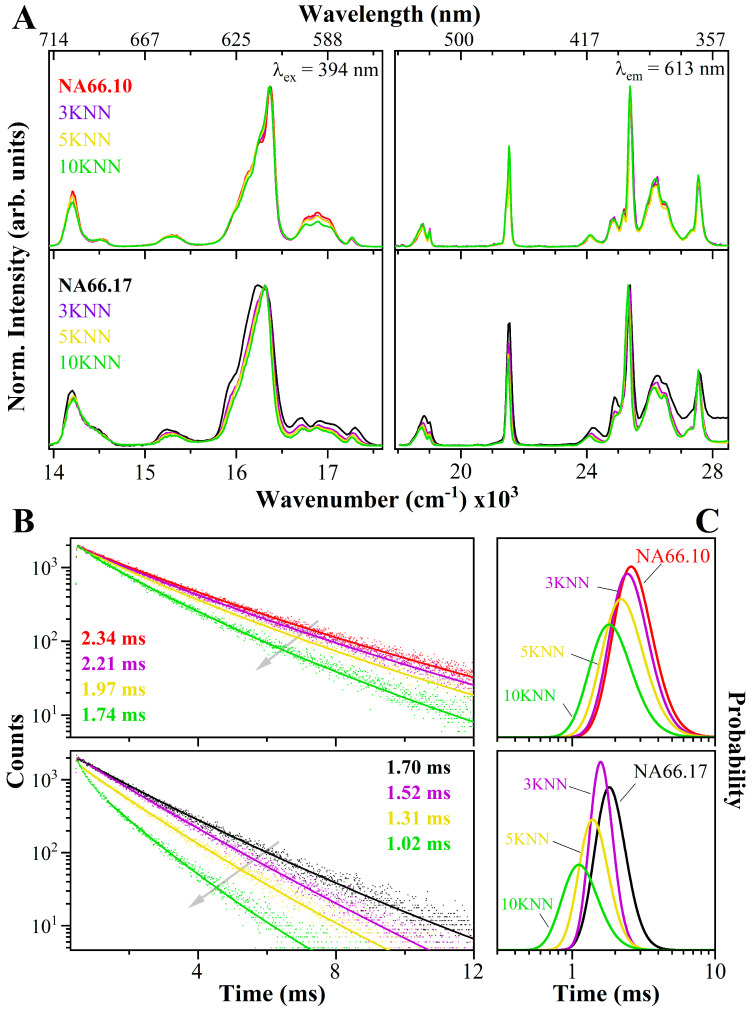
(**A**) Photoluminescence emission and excitation, and (**B**) fluorescence decay curves at 613 nm (all under 394 nm excitation) of the two Eu-doped glass series having increasing KNN contents. By adding KNN, both series show variations in the Eu^3+^ local environment and a decrease in the Eu^3+^ decay lifetime. A mono-exponential decay is associated with 3 mol% KNN contents in both series, whereas an additional shorter component occurs for 5 and 10 mol% KNN contents. (**C**) The lifetime probability distributions are reported for all glasses.

The photoluminescence emission and excitation spectra for the two Eu-doped glass series having increasing KNN contents are reported in Figure 6A (respectively collected at the maximum of the excitation, λ_ex_ = 394 nm, and of the emission, λ_em_ = 613 nm). All the sharp peaks are associated with Eu^3+^ transitions, and no additional broad bands, related to reduced Eu^2+^ species can be observed (see also Figure S3).

The EM spectra in the NA66.10 series show only minor changes in the transitions upon the addition of KNN. Noteworthy is the lower relative intensity of the $F_{1}$ transition, which results in a slight increase in the asymmetricity ratio $R_{2/1}$ (see Table 2), indicating a higher distortion degree of the local environment where the Eu^3+^ ions are accommodated. On the contrary, with the increase in KNN, samples of the NA66.17 series show very clear changes in the emission spectra, with strong modifications of the $F_{2}$, $F_{1}$, and $F_{0}$ bands, and a net decrease in the $R_{2/1}$. In particular, upon the addition of KNN, there is a clear decrease in the energy splitting of the Stark components of the $F_{1}$ and $F_{2}$ transitions, and the shift to lower energies of the $F_{0}$ band along with a decrease in its width and asymmetricity. Indeed, the latter shows a large decrease in the FWHM, from ~115 cm^−1^ in the pristine glass to about 1.5 times narrower in the sample with 10 mol% KNN (~78 cm^−1^).

To assess the observed changes, mainly in terms of the local crystal field strength, the second-rank crystal field parameter ($B_{20}$) was considered. This parameter has already been used in both crystalline materials and sodium aluminosilicate glasses to study the variations in the Eu^3+^ transitions [32,37,38]. In particular, this parameter is well suited for glasses, because it is non-zero only in low-symmetry environments, as is typically the case with Eu^3+^ local environments within amorphous materials [30]. The second-rank crystal field parameter $B_{20}$ was calculated according to equation [2], and considers the integrated intensities *I* and frequency positions (as barycenter) of the $F_{2}$ and $F_{0}$ bands [32,37,38]:

(2) I(D0 →5 F07)I(D0 →5 F27)=4(B20)275(∆20)2

where ${∆}_{20}$ represents the energy separation between the barycenters of the $F_{2}$ and $F_{0}$ transitions (cm^−1^). According to [32,38], the transition strength of the $F_{0}$ band is proportional to the $B_{20}$, whereas the $F_{2}$ line is independent. Ergo, the increase in $B_{20}$ is associated with an increase in crystal field perturbation. The crystal field parameter in the two glass series (Table 2) shows an opposite trend, in agreement with the observations performed on the asymmetricity ratio (plotted in Figure S4). In the NA66.10 glass series, $B_{20}$ linearly increases by adding KNN, moving from ~425 to ~500 cm^−1^. In the polymerized NA66.17 glass series, the strong variations in the $F_{0}$ band are mirrored in the drastic drop of $B_{20}$ that goes from ~680 cm^−1^ down to ~450 cm^−1^ by increasing KNN content.

From these observations, it is possible to conjecture that the stronger variations in the Eu^3+^ emission spectral lines occurring in the NA66.17 series upon the addition of KNN indicate that Eu ions preferentially move in the proximity of the [NbO_6_] species, leading to a consequent lower distortion degree of the Eu site and a weaker crystal field. On the contrary, in the peralkaline glass NA66.10, there is a more homogeneous distribution of the Eu^3+^ ions, most likely connected to both niobate and silicate structural units.

Fluorescence lifetime measurements were carried out on the most intense Eu^3+^ emission line (λ_em_ = 613), under excitation of 394 nm (Figure 6B). The polymerized KNN-doped NA66.17 glasses all have a shorter lifetime than the peralkaline ones, and the decay lifetime is systematically reduced in both glass series with an increase in KNN content. Samples containing 3 mol% KNN exhibit a straight line, indicating a mono-exponential decay. The increase in KNN causes a steeper slope (shorter lifetime) and a more pronounced bend, particularly in the 10 mol% KNN samples. Consequently, the decay comprises more than one exponential component. The mono-exponential part was used to extrapolate the lifetime probability distributions shown in Figure 6C. A shorter component, estimated to be about 0.2 ms, is clearly present in NA66.17_10KNN.

The systematic decrease in the lifetime in both series may be correlated with the distortion of the host site, resulting from the relaxation of the selection rules governing the forbidden *f–f* transitions [39]. However, in this particular case, it is considered more appropriate to take into account the change in the refractive index n_D_ of the glasses, which exhibits a strong linear correlation with fluorescence lifetime (see Figure S4). In fact, the interaction between the dielectric medium and the photoemitter ions may be linked to the increase in the spontaneous emission rate, and the subsequent decrease in the excited state’s lifetime [40,41].

The Judd–Ofelt (JO) intensity parameters Ω_λ_ (λ = 2, 4, 6) were evaluated from the PL emission spectra of all Eu-doped glasses under 393 nm excitation. The values of Ω_2_, Ω_4_, and Ω_6_ were determined by means of the integrated intensities of the emission D0 →5F2,4,67 lines following the previous reports [30,33,37] (details in the Supplementary Materials), and are listed in Table 2, along with the calculated radiative lifetime (τ_r_). The intensity parameters Ω_λ_ in the pristine glasses follow the trend observed in other aluminosilicate glasses, with Ω_2_ > Ω_4_ > Ω_6_ ([37] and references therein). By adding KNN, the Ω_2_ parameter increases in both series with respect to the pristine glasses. Since this parameter can be related to “short-range order effects” [42] such as the bond covalency character and the local symmetry, the upward tendency confirms a different environment and variations in the bond distances around the Eu^3+^ ions in an alkali-niobate-bearing matrix. In particular, the polymerized series, upon the addition of KNN, shows a stronger increase in the Ω_2_ parameter, and, in turn, a stronger variation in the covalency of the ligand field surrounding the Eu^3+^ ions. This result agrees well with the observations already performed using $R_{2/1}$, and $B_{20}$.

#### 3.2.3. Eu-Doped Glass-Ceramics

**Figure 7:**
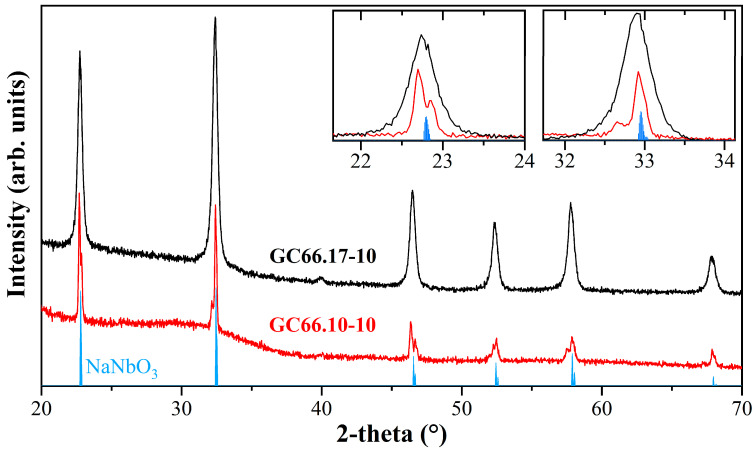
XRD patterns of the two GCs samples. The main reflections of orthorhombic NaNbO_3_ (JCPDS. 33-1270) are reported for comparison (blue line). The GC samples show a shift in the reflections to smaller diffraction angles compared to NaNbO_3_, in agreement with the incorporation of K^+^ ions into the crystalline structure. The broadening in GC66.17-10 is related to the crystallization of sub-micron crystals.

Glass-ceramic (GC) samples obtained from the glasses having 10 mol% KNN show the presence of a single crystalline phase. Sharp reflections are visible in the diffraction pattern of the peralkaline GC (sample label GC66.10-10), while the NA66.17-10KNN glass-ceramic (labeled GC66.17-10) shows much broader reflections, although it has similar *d*-spacing (Figure 7). Both GCs have reflections similar to those of the orthorhombic NaNbO_3_ phase (space group *Pbma*, JCPDS. 33-1270), but not identical, because of the evident shift to smaller diffraction angles, in agreement with the incorporation of K^+^ ions into the crystalline structure.

The shape of the reflections agrees with the different crystallization mechanisms observed for the two bulk compositions. Indeed, the glass-ceramic obtained from sample NA66.10-10KNN shows preferential surface crystallization with crystals propagating from the edge towards the center. A holding time of 2 h at 900 °C allowed the formation of visible surface crystals and the preservation of an internal amorphous bulk matrix, evidenced by the strong amorphous bell in the diffraction pattern. The GC66.17-10 glass-ceramic sample, on the other hand, shows volume crystallization of sub-micron crystals, allowing a high degree of transparency even after longer thermal treatments (4 h). In the latter, the small size of the crystals is responsible for the broadening of the reflections in the XRD pattern. The Le Bail analysis determined the lattice parameters, the unit cell volume, and the crystallite size, as reported in Table 3. Although in a Na^+^-rich environment, the larger K^+^ ions enter the perovskite structure, causing an increase in unit cell volume. This indicates preferential K^+^ partitioning into the crystal phase and the possibility of adjusting the K/Na ratio of the glass matrix to achieve specific K_x_Na_(1−x)_NbO_3_ phases.

**Figure 8:**
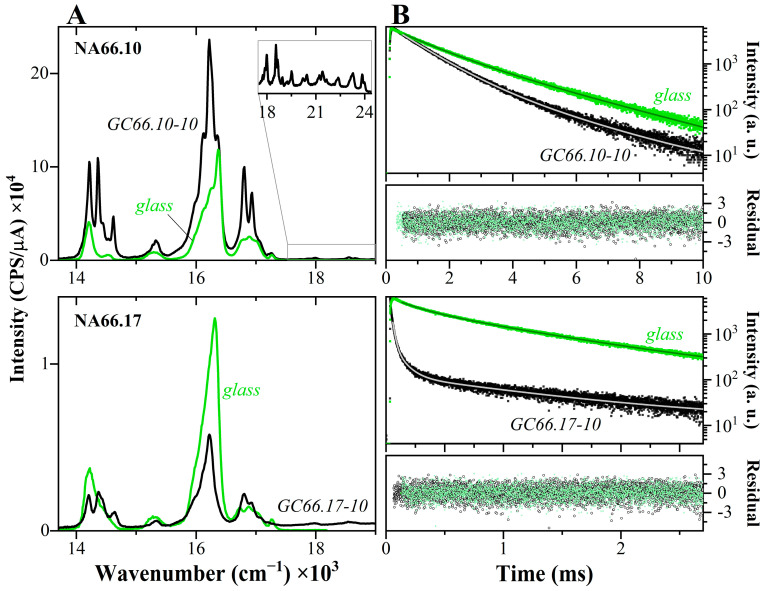
Photoluminescence emission (**A**) of the two glass series having 10 mol% KNN contents and the corresponding GCs, and fluorescence decay curves (**B**) at 613–616 nm (all under 394–397 nm excitation). The upper panels are related to the peralkaline composition NA66.10, and the lower panels to the polymerized NA66.17. The inset in (**A**) highlights the transitions from higher excited levels, visible in both GCs. In (**B**), the decay curves show the differences between the glasses and the corresponding crystallized samples. In the GCs, there is a decrease in the Eu^3+^ decay lifetime, and the increasing contribution from a faster component.

The photoluminescence behavior of the two GC samples is also quite different, and some major differences in the emission (Figure 8A) and excitation (Figure S5A) spectra can be highlighted:

- The excitation spectra of the GC66.17-10 sample (Figure S5A) show a strong change in the relative intensities of the Eu^3+^ main transitions, with the F0 →7D25 and F0 →7D15 lines (respectively at 21,510 cm^−1^ and 18,845 cm^−1^) having a much higher relative intensity compared to the parent glass. A more detailed discussion of the different excitation lines in this sample is reported below.
- The peralkaline GC shows a higher emission intensity than the parent glass, whereas the crystallized samples from the polymerized compositions (GC66.17) show a strong decrease in emission intensity. The reduced intensity in the latter sample is partially caused by the occurrence of reduced Eu^2+^ species, attested by the presence of the broad excitation and emission bands centered respectively at ~26,920 cm^−1^ and 21,475 cm^−1^ (Figure S5B).
- In both GCs, the FJ7 transitions become structured and narrow, and show strong Stark splitting into two or more distinct bands, showing the partial incorporation of Eu^3+^ ions into the crystalline phase.
- GCs show weak contributions at higher frequencies (inset in Figure 8) associated with the emission from higher excited D1−35 levels [30,43], supporting the conclusion that Eu^3+^ ions are incorporated into a lower phonon energy environment (such as the niobate crystals), reducing non-radiative energy relaxation.
- The F07 transition in the peralkaline GC66.10 is split into two components, indicating the presence of (at least) two distinct populations. Remarkably, this band is not clearly visible in the sample GC66.17-10. Since this transition is an indicator of site symmetry, it can be concluded that in the transparent GC66.17-10, the Eu^3+^ ions occupy sites with a higher degree of symmetry.
- The fluorescence lifetime (Figure 8B) decreases in both GCs compared to the parent glasses, and the two GCs decay curves show the occurrence of fast and long decay components. A double exponential function appropriately describes the decay curves collected at the maximum of the emission band (λ_em_ = 616 nm), resulting in a lifetime of 1.89 ± 0.07 ms and 0.86 ± 0.01 ms, respectively, for long and fast decay of the GC66.10 sample. The polymerized GC66.17 has a complex decay with a prominent fast decay, and the best fit that provided randomly distributed residuals, and a χ^2^ of 1.1 was obtained with three exponential decay components. The results indicate the presence of two short contributions (~20 μs and 90 ± 4 μs) and a longer one of 1.12 ± 0.03 ms. In both GCs, the long decay component resembles the ones in the parent glasses, suggesting that some of the Eu ions are still in the residual glass. Note that the shortest lifetime value estimated, here, must be carefully considered, because of the limitation of the setup used to collect the data (Xe-lamp).

**Figure 9:**
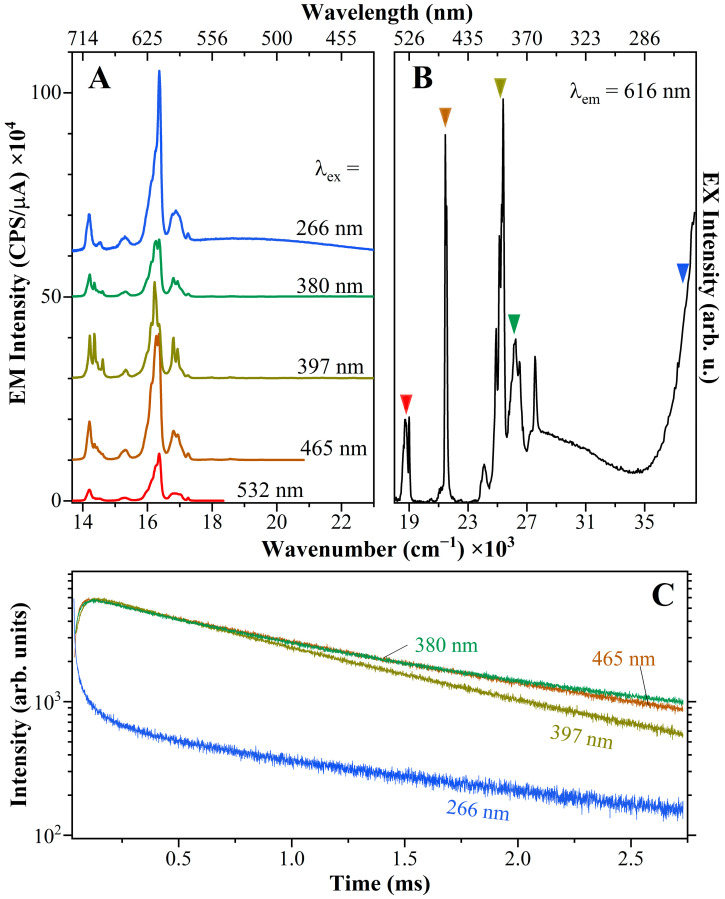
Excitation-dependent emission spectra (**A**) of the GC66.10-10. The positions of the excitation lines used are shown in (**B**). Noticeable differences in the photoluminescence emissions can be observed depending on the excitation wavelength. The fluorescence decay data are reported in (**C**) by monitoring the λ_em_ = 616 nm. All differences shown here point to the occurrence of distinct luminescence mechanisms.

As previously stated, the peralkaline glass-ceramic (GC66.10-10) shows a split of the electric-dipole D0 →5F07 transition in the emission spectrum, as well as strong relative intensities of the two Eu^3+^-related transitions in the excitation spectrum. To gain a better understanding of the population distribution in this sample, excitation-dependent emission spectra, and fluorescence decay data were collected (monitoring for all the λ_em_ = 616 nm) (Figure 9). A noticeable difference was observed, particularly when using excitation wavelengths of 266 and 532 nm, which gave a broader photoluminescence emission profile that is characteristic of an amorphous material. Additionally, the emission spectrum excited at 266 nm shows the occurrence of a broad band centered around 18,900 cm^−1^, which is not related to the presence of reduced species, such as in the case of the polymerized composition, but instead could be related to the energy transfer from host material to Eu^3+^ (see Section 3.3). From the excitation-dependent emission spectra, besides the different splitting of the Stark components of the emission lines, it is also possible to observe variations in the broadening of D0 →5F07 transition and in the relative intensity between the D0 →5F17 and →F27 transitions. The calculated asymmetric ratios for these spectra vary from 3.7 (under 397 nm excitation) to 5 (under 266 nm excitation) and to 5.7 (under 465 nm excitation).

Usually, when any energy level above the D05 is stimulated, there is a fast non-radiative relaxation to that energy level. As a result, the same photoluminescence emission is detected regardless of the excitation line used. Therefore, in the case of the GC66.10-10 sample, the significant differences observed suggest the involvement of distinct luminescence mechanisms, likely involving energy transfer (including both the host emission and resonant energy transfer) and migration processes [44,45]. In particular, the detection of broader spectra (amorphous-like) under certain excitation lines suggests the occurrence of resonance transfers from the Eu^3+^ species incorporated in the crystal phases to those still present in the residual glass matrix, or vice versa, from the glass to the crystalline phase. The fluorescence decay curves show well the differences depending on the excitation line used, supporting the presence of different Eu populations and different photoluminescence mechanisms (Figure 9C). Nevertheless, the study of the shortest components with the Xe lamp used here is challenging. To clarify the main mechanisms, further studies could use fluorescence line narrowing and site-selective fluorescence decay under different excitation lasers [46].

### 3.3. Rare Earth-Doped Self-Activated Glass Phosphor

In the literature, there are many studies on the photoluminescence behaviors of rare earth-doped self-activated phosphors based on niobate crystals, and a recent review was provided by [47]. Crystalline niobate hosts, such as Ca_2_Nb_2_O_7_, CaNb_2_O_6_, CdNb_2_O_6_, YNbO_4_, and LaNbO_4_, have been studied for applications such as self-luminescent complexes, quantum-cutting luminescent solar concentrators, and for their tunable properties, with very promising results according to the literature [47,48,49,50,51]. On glasses, to the best of our knowledge, only [25] reported the occurrence of an energy transfer process from the niobate to lanthanide ions in a 24La_2_O_3_–10Nb_2_O_5_–65B_2_O_3_:1Eu_2_O_3_ glass. In the present work, to verify if the niobo-aluminosilicate glasses could be used as potential lanthanide ion-activated luminescent materials, the Eu-doped samples proving to have the strongest luminescence (peralkaline glasses NA66.10_3KNN and _5KNN) were considered.

**Figure 10:**
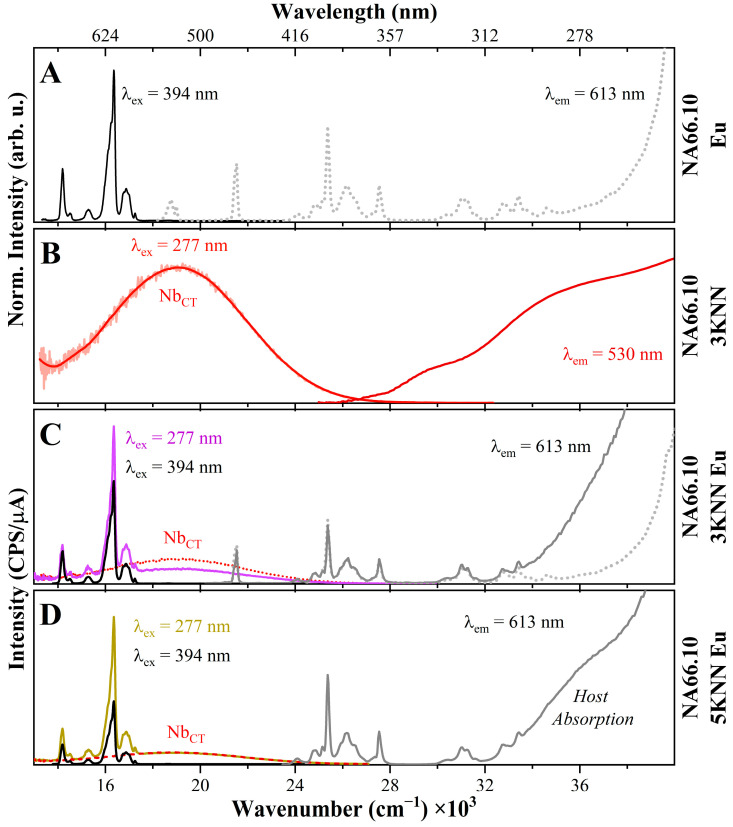
Room temperature photoluminescence emission and excitation spectra for glasses in the NA66.10 series (exit and entrance slits = 1 nm). The Eu-doped pristine glass (**A**) shows sharp Eu^3+^ transitions, while the sample having 3 mol% KNN (**B**) has broad bands related to the O^2−^ → Nb^5+^ charge transfer (Nb_CT_), with an emission centered around 19,400 cm^−1^. In the lower panels, the NA66.10 glasses, containing Eu and 3 mol% (**C**)/5mol% KNN (**D**), show the occurrence of both Eu^3+^ and Nb_CT_ transitions. Indeed, under an excitation of 277 nm (purple curve), the broad Nb_CT_ is visible and perfectly overlaps with the one in the undoped glass (dotted red line, reported for comparison), and the Eu^3+^ emission intensity increases, confirming that an energy transfer from the host [NbO_6_] to the Eu^3+^ ions has occurred.

From the results reported in the previous sections, it was observed that the Eu-doped pristine peralkaline glass (NA66.10) has only electronic transitions related to Eu^3+^ ions, with very sharp peaks (Figure 10A). No broad bands related to reduced Eu^2+^ species were observed, and the rise at a very high frequency (above 39,000 cm^−1^) is due to the correction of the signal to the Xe lamp intensity. When adding KNN to the pristine glass, it was observed that the peralkaline glass containing 3 mol% KNN (NA66.10_3KNN) had the strongest PL emission, with a broad band centered around 18,100 cm^−1^ upon excitation at 277 nm (example in Figure 10B). The other peralkaline sample with 5 mol% KNN showed a similar broad band, but with a much lower intensity. These emission bands are related to the charge transfer transition of electrons from O^2−^ to Nb^5+^ ions (Nb_CT_), as discussed in Section 3.1.

The samples containing Eu and KNN, as previously observed, show the occurrence of sharp Eu^3+^ transitions when excited at the maximum of the Eu^3+^ excitation lines (394 nm), but no additional broad bands (Figure 10C,D). However, if excited in UV, under wavelengths matching the host absorption (277 nm), besides the sharp Eu^3+^ lines, a broad emission centered around 18,500 cm^−1^ is visible. This wide band perfectly resembles the Nb_CT_ emission observed in the parent niobate sample (reported as a dotted red line for comparison in Figure 10C,D). Furthermore, it is clear that there is an increase in Eu^3+^ emission intensity in the Eu-doped niobo-aluminosilicate glasses. The pristine glass, when excited under UV light, shows very weak Eu^3+^ lines, and only in the niobate glasses is there a strong enhancement of PL emission intensity (see Figure S6). A similar mechanism was observed in the GC sample reported in Figure 9, where a UV excitation caused the strongest Eu^3+^ emission intensity and the occurrence of the Nb_CT_ contribution at ~18,900 cm^−1^. These observations indicate that an energy transfer from the host [NbO_6_] to Eu^3+^ occurs, therefore validating the possibility of exploring niobate glasses as REE activators. Niobium-based glasses and glass-ceramics offer greater versatility, compared to crystalline materials, both in terms of achievable chemical variability and because the intensity and positions of the main bands will vary depending on the nature of the host structure. Furthermore, the low phonon energy of the Nb-based host enhances PL efficiency. Therefore, they are promising candidates for novel lanthanide ion-activated luminescent materials. The encouraging results observed here justify further investigations. In fact, additional studies will be conducted on other rare earth elements since the broad emission bands overlap with the excitation bands of several lanthanide ions. Furthermore, these studies will consider the energy transfer mechanisms in glass-ceramics (GCs) containing piezoelectric crystals. This could have many applications due to the potential triboluminescence effects caused by pressure-induced electron transfer to the host material and subsequently to the dopant.

## 4. Conclusions

Two glass series with different polymerizations and containing alkali niobates from 0.4 mol% up to 20 mol% were investigated with the aim of providing a systematic study of the photoluminescence and fluorescence lifetime of niobium in alkali-niobate aluminosilicate glasses. It was found that under UV light excitation, a charge transfer transition of electrons from O^2−^ to Nb^5+^ ions gives rise to an intense visible emission. However, the emission intensity is strongly dependent on the niobate content because the connectivity of the [NbO_6_] units changes. A higher fraction of distorted [NbO_6_] units is able to enhance the luminescence. A relationship between the Stokes shifts and the structural environment was established, allowing the distortion of the Nb local environment to be deduced from this parameter.

In order to verify if niobate glasses could be used as potential REE activators, samples of the two series were doped with Eu_2_O_3_. The analysis of both photoluminescence and fluorescence lifetime allowed us, first of all, to observe the strong influence of niobate content on Eu^3+^ crystal field strength and to gain more insight into the structural evolution of the glass network. Second, the occurrence of an energy transfer from the host [NbO_6_] to Eu^3+^ was observed, opening up the possibility of exploiting niobate glasses and glass-ceramics as efficient REE ion-activated luminescent materials.

The crystallization of the samples shows the successful development of alkali-niobate perovskite glass-ceramics (GCs). The samples have different crystallization and luminescence behaviors depending on the bulk composition, and in the polymerized composition, transparent GCs were obtained. Also, depending on the bulk chemistry, the Eu^3+^ distribution seems to be different, with at least one population in the matrix and one in the crystalline phase. In fact, the excitation-dependent emission in the peralkaline glass indicates energy transfer and migration of electronic excitation from one Eu population to another. All the results presented here indicate that the use of niobate-based glasses and glass-ceramics has great potential and could find a wide range of applications in sensors, solid-state lasers, and up-conversion luminescent materials, in the development of optoelectronic devices and for photo-electro-mechanical coupling.

## Figures and Tables

**Table 1:** List of the samples investigated and their Nb_2_O_5_ content (chemical analysis from [11]). The results of the photoluminescence and optical absorption study are reported: the frequency position of the absorption edge (Abs-edge), emission (EM), and excitation (EX) bands, along with the Stokes shifts (all data acquired with slits width of 3 nm).

Label	KNN Content (mol%)	Nb_2_O_5_ ^$^ (mol%)	Max EM (cm^−1^) ±50 cm^−1^	EM Intensity (CPS/μA)	EM FWHM (cm^−1^)	Max EX (cm^−1^) ± 250 cm^−1^	Abs-Edge (cm^−1^) ^$^	Stokes Shifts (cm^−1^)
NA66.10_								
0.5KNN	0.4	0.19	18,500	2.17 × 10^5^	5738	35,800	35,436	17,300
1KNN	0.8	0.38	18,200	3.10 × 10^5^	6050	35,400	34,748	17,200
3KNN	2.9	1.45	18,100	3.26 × 10^5^	6391	35,000	33,799	16,850
5KNN	5.4	2.70	18,350	1.25 × 10^5^	6235	34,600	32,831	16,250
10KNN	10	4.73	18,600	7.29 × 10^4^	6025	34,200	32,468	15,600
20KNN	20	9.97	18,550	7.38 × 10^3^	4508	33,500	31,167	14,950
NA66.17_								
0.5KNN	0.4	0.19	19,250	1.50 × 10^5^	5422	36,500	35,211	17,300
1KNN	0.8	0.38	19,400	2.43 × 10^5^	6105	36,000	34,843	16,600
3KNN	2.9	1.68	19,400	1.10 × 10^5^	6375	34,800	33,581	15,400
5KNN	5.4	2.88	19,300	3.20 × 10^4^	6154	33,700	32,282	14,400
10KNN	10	4.83	19,350	1.38 × 10^4^	6368	*-*	30,532	-

$ data from [11].

**Table 2:** The asymmetricity ratio $R_{2/1}$, the second-rank crystal field parameter $B_{20}$ (cm^−1^), the experimental fluorescence lifetime (τ_exp_, ms), the results from the Judd–Ofelt analysis (Ω_λ_ × 10^−20^ cm^2^ and τ_r_, ms), and the calculated quantum efficiency (η, %) are reported (see also Supplementary Materials). Refractive index values are from [11].

Sample	$R_{2/1}$	$B_{20}$ cm^−1^	τ_exp_ ms	τ_r_ ms	η %	Ω_2_ × 10^−20^ cm^2^	Ω_4_ × 10^−20^ cm^2^	Ω_6_ × 10^−20^ cm^2^	n_D_
NA66.10_Eu	4.4	425	2.34(1)	2.92	75%	7.3(2)	2.3(3)	1.4(1)	1.503(1)
3 KNN_Eu	5.0	440	2.21(1)	2.4	76%	7.4(2)	2.3(2)	1.5(2)	1.5242(6)
5 KNN_Eu	5.2	462	1.97(1)	2.3	73%	7.6(3)	2.4(2)	1.7(2)	1.545(1)
10 KNN_Eu	6.3	506	1.74(2)	2.15	73%	8.5(3)	2.5(1)	1.1(1)	1.5732(5)
NA66.17_Eu	14.7	687	1.70(3)	2.6	63%	8.8(2)	2.8(7)	2.2(3)	1.494(2)
3 KNN_Eu	13.0	587	1.52(1)	1.64	76%	11.5(4)	3.8(1)	2.4(2)	1.517(1)
5 KNN_Eu	12.1	538	1.31(1)	1.55	69%	11.4(3)	4(2)	3(2)	1.542(2)
10 KNN_Eu	11.2	458	1.02(3)	1.42	60%	12(4)	4.4(4)	2.3(3)	1.589(1)

**Table 3:** Synthesis conditions for the GC samples, unit cell parameters (Å), volume of the cell (V, Å^3^), and crystallite size (nm) of the crystallized phases.

Label	T Crystallization (°C)	Holding Time (h)	Space Group	*a, b, c* (Å)	*V* (Å^3^)	Crystallite Size (nm)
GC66.10-10	900	2	*Pbcm* (*Pbma*)	5.56301/ 15.56069/ 5.51447	477.36	119
GC66.17-10	900	4	*Pbcm* (*Pbma*)	5.54292/ 15.60753/ 5.51829	477.39	28

## Data Availability

Data are contained within the article and Supplementary Materials. Further inquiries can be directed to the corresponding author [M.R.C.].

## Supplementary Materials

The following supporting information can be downloaded at https://www.mdpi.com/article/10.3390/ma17102283/s1. Figure S1: PL emission spectra collected under 275 nm excitation with different entrance and exit slit widths; Figure S2: Corrected and uncorrected PL excitation spectra; Figure S3: PL excitation and emission spectra of the Eu-doped KNN-bearing glasses; Figure S4: Variation in the Eu local environment depending on the KNN content; Figure S5A: PL of the Glass-ceramics; Figure S5B: Photoluminescence emission and excitation spectra of the Eu-doped NA66.17 pristine glass, the glass NA66.17 with 10 mol% KNN, and the corresponding GC; Figure S6: Photoluminescence emission and excitation spectra for the Eu-doped pristine glass NA66.10, the NA66.10-5KNN glass, and the same glass composition containing both Eu and 5 mol% KNN. Appendix: Judd–Ofelt analysis for Eu^3+^ emission. Refs. [52,53,54,55,56,57,58,59] are cited in the supplementary materials.
