# Supplementary material for: Photoluminescence Study of Undoped and Eu-Doped Alkali-Niobate Aluminosilicate Glasses and Glass-Ceramics
Source: Materials (Basel). 2024 May 11;17(10):2283. doi: 10.3390/ma17102283 (PMC11122913; doi:10.3390/ma17102283)
Supplement: Supplementary file 1 [file materials-17-02283-s001.zip › materials-2946581-supplementary.pdf]

materials

MDPI

Supplementary Materials

Photoluminescence Study of Undoped and EU-Doped Alkali-Niobate Aluminosilicate Glasses and Glass-Ceramics

Maria Rita Cicconi [1,*], Hongyi Deng [1], Takahito Otsuka [2], Aadhitya Telakula Mahesh [1], Neamul Hayet Khansur [1], Tomokatsu Hayakawa [2] and Dominique de Ligny [1]

1 - Friedrich-Alexander-Universität Erlangen-Nürnberg, Department of Materials Science and Engineering, Institut für Glas und Keramik, Martensstrasse 5, 91058 Erlangen, Germany
2 - Nagoya Institute of Technology, Department of Life Science and Applied Chemistry, Gokiso-cho, Showa-ku, Nagoya 466-8555, Japan
* Correspondence: maria.rita.cicconi@fau.de, Tel: +49 9131 85-27555

Figure S1 – PL emission spectra collected under 275 nm excitation with different entrance and exit slit widths of the glasses NA66.10-1KNN and NA66.10-3KNN

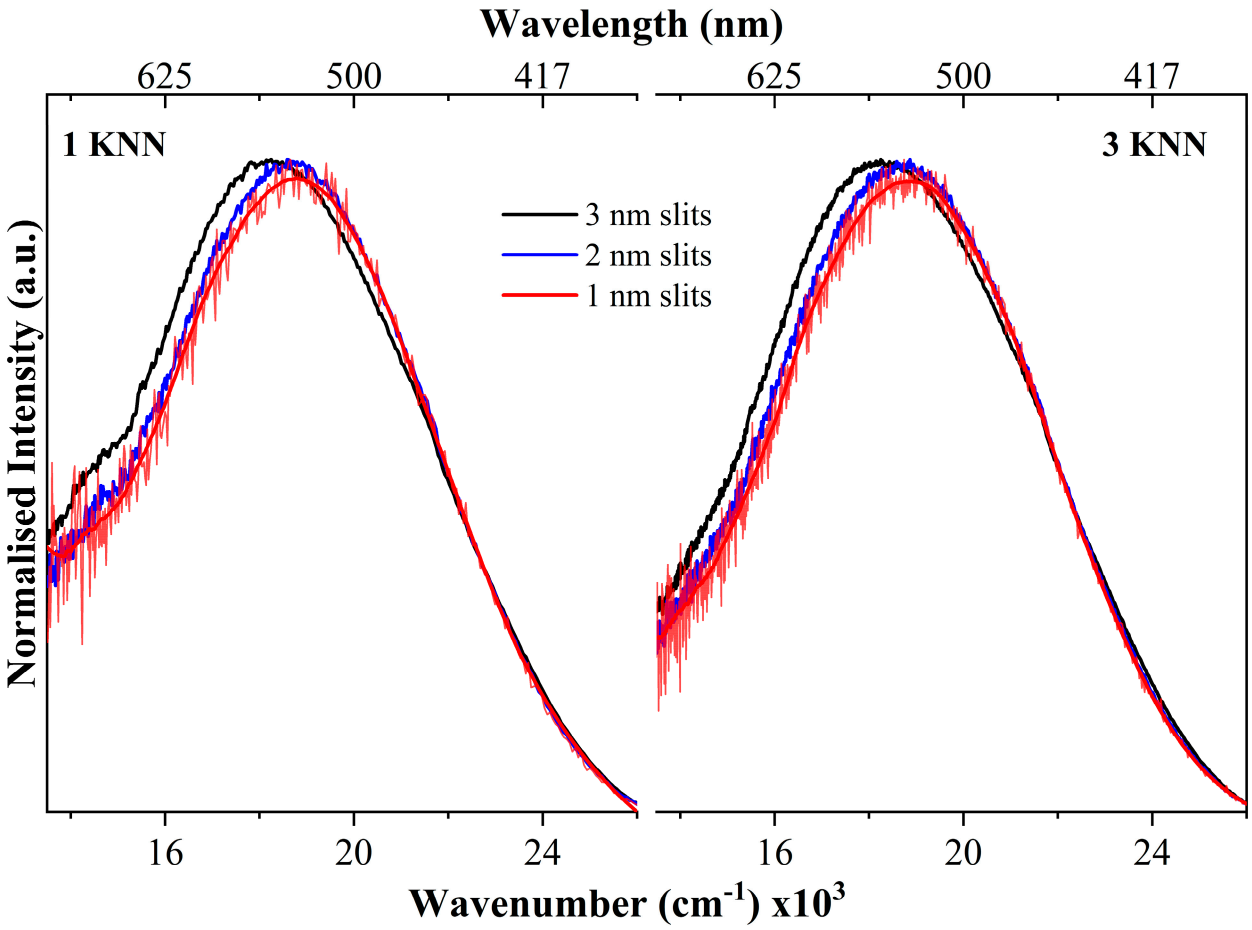

Figure S1. Photoluminescence emission spectra of the glasses in the series NA66.10 having 1 and 3 mol% KNN. The spectra were collected under 275 nm excitation with different entrance and exit slit widths: from 1 to 3 nm (the bands have been normalized to the maximum intensity for comparison). The whole emission band becomes narrower by decreasing the slit size, and in particular, the shoulder at lower frequencies (~ 16400 cm^{-1}) becomes less important.

Figure S2 – Corrected and uncorrected PL excitation spectra.

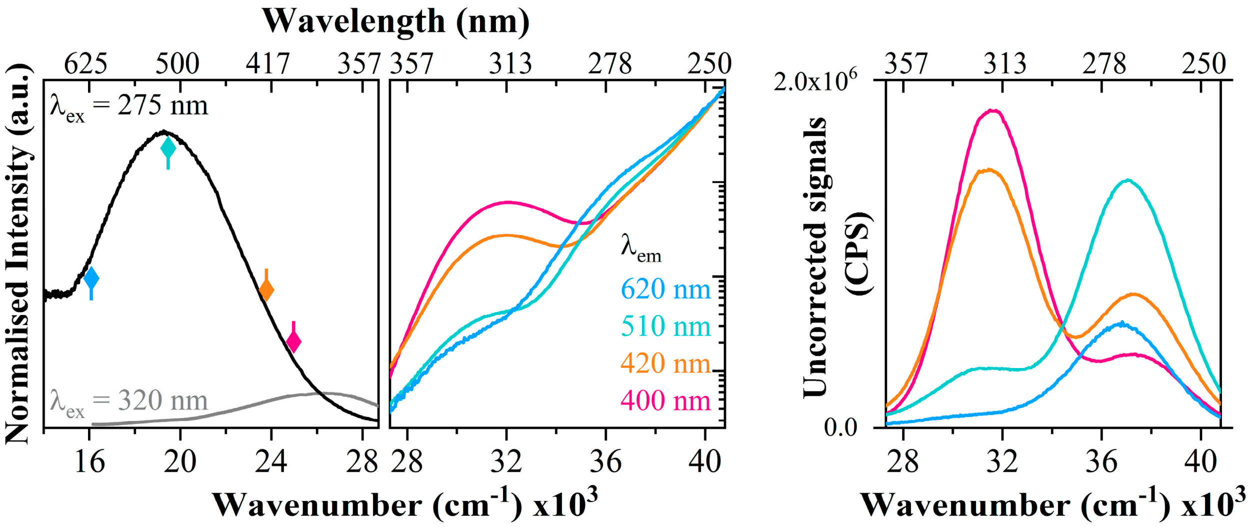

Figure S2. Excitation spectra were collected at different positions along the emission band (400 nm, 420 nm, 510 nm, and 620 nm) for the glass NA66.10 containing 1 mol% KNN. Depending on the emission wavelength selected, the relative intensities of the two excitation bands change. On the right panel, the lamp-uncorrected signals are reported. Despite the relative intensities of the two contributions cannot be compared in the uncorrected signals, it is clearly visible that the two excitation centers strongly depend on the emission wavelength. This suggests that at least two different populations are responsible for the emission.

Figure S3 – PL EX and EM of the Eu-doped KNN-bearing glasses

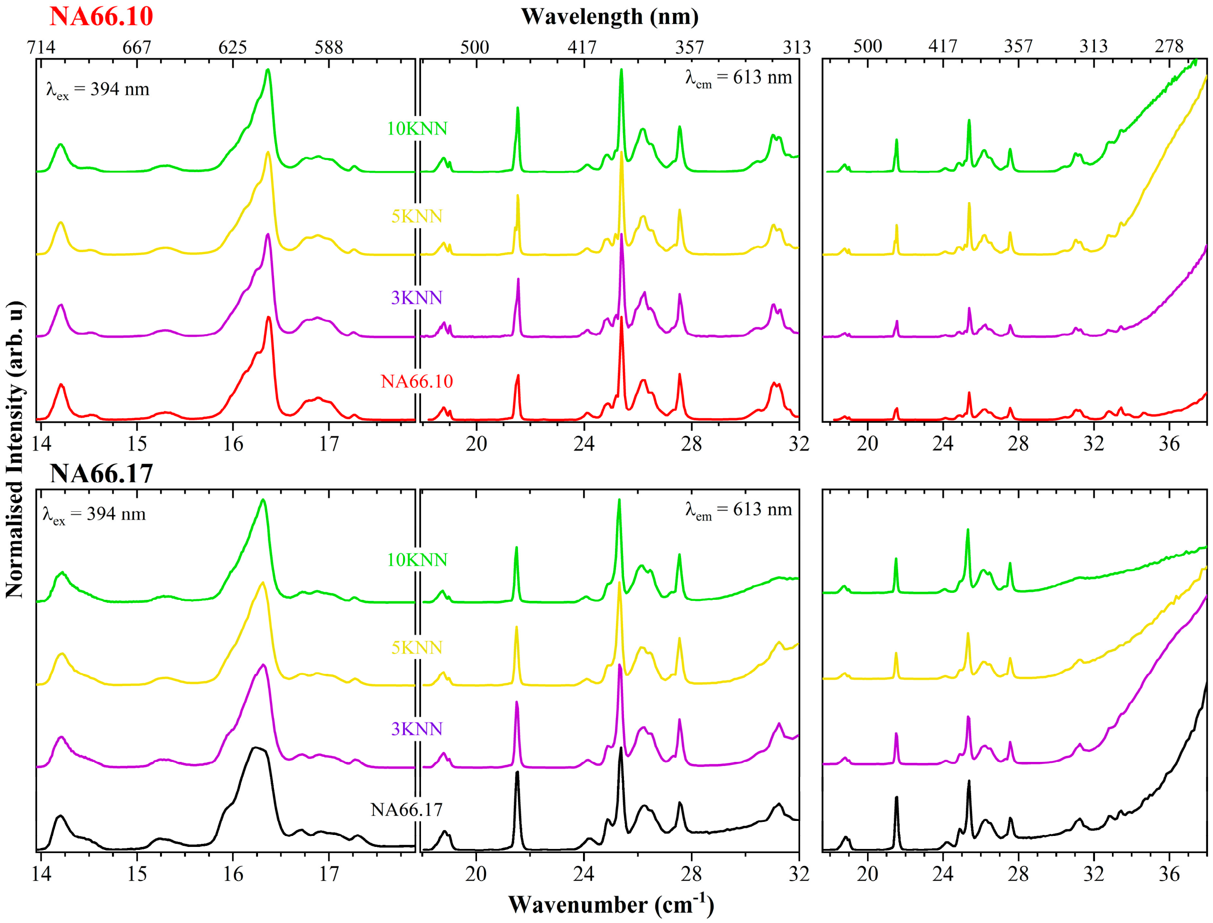

Figure S3. Photoluminescence excitation (center and right panels) and emission spectra (left panel) of the Eu-doped glasses in the NA66.10 and NA66.17 glass series. The whole frequency range of the excitation spectra is shown in the right panel.

Figure S4 - Variation of the Eu local environment depending on the KNN content. Correlation of the lifetime with the refractive index of the medium.

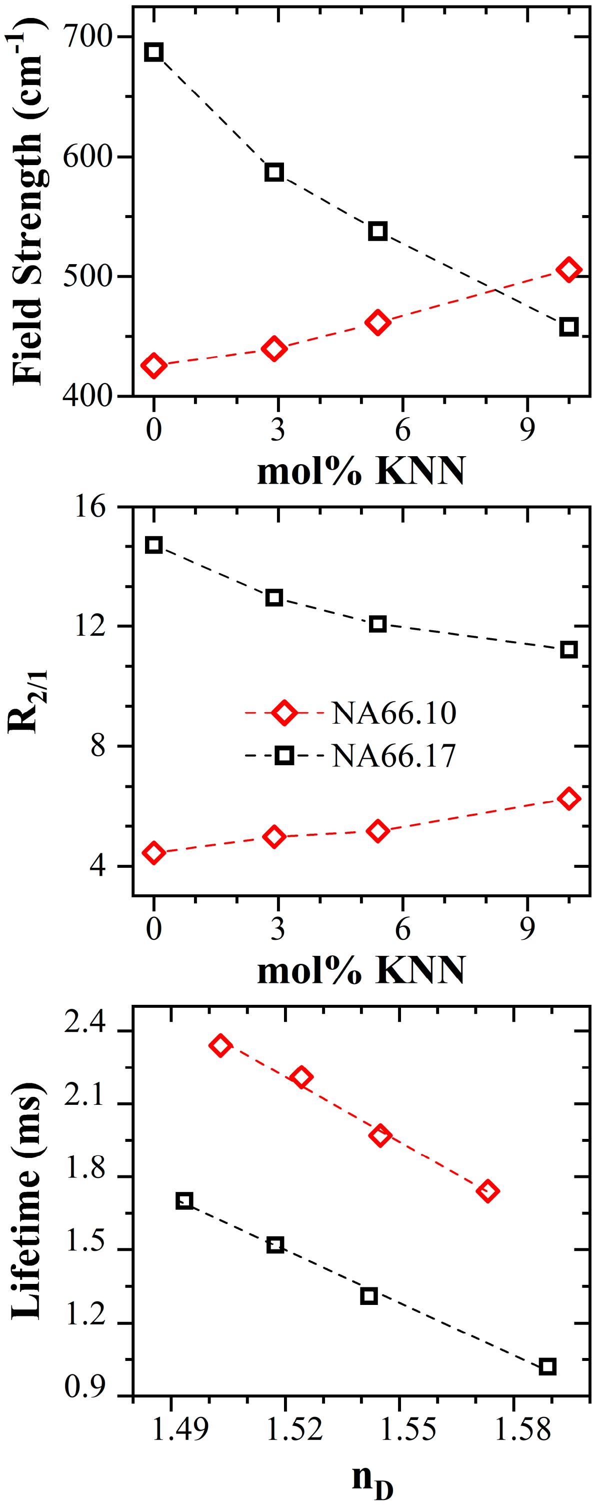

Figure S4. Variation of the Eu local environment depending on the KNN content is represented by the variation of the Field Strength (B_{20} parameter) and the asymmetricity parameter ($R_{1/2}$). The refractive index of the glasses increases by increasing KNN (see Table 2). In the lower panel, it can be observed the linear correlation of the Eu^{3+} fluorescence lifetime with the refractive index of the medium.

Figure S5 – PL of the Glass-ceramics

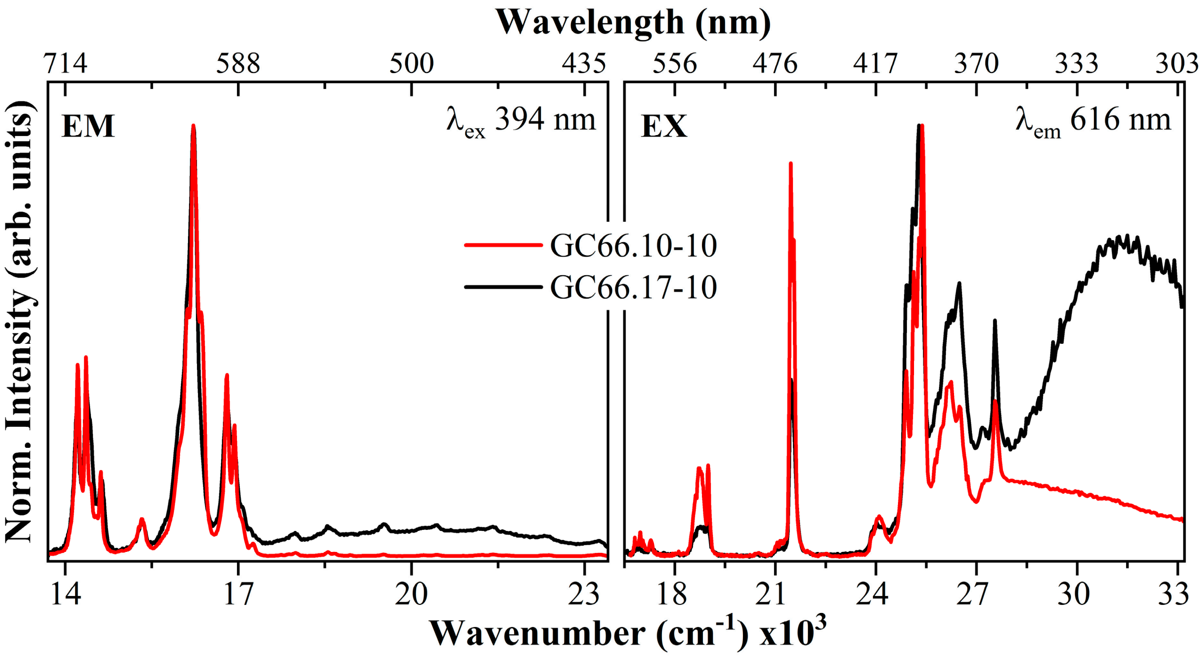

Figure S5. A: Photoluminescence emission (left panels) and excitation (right panels) spectra of the Eu-doped GCs in the NA66.10 (red lines) and NA66.17 (black lines) series.

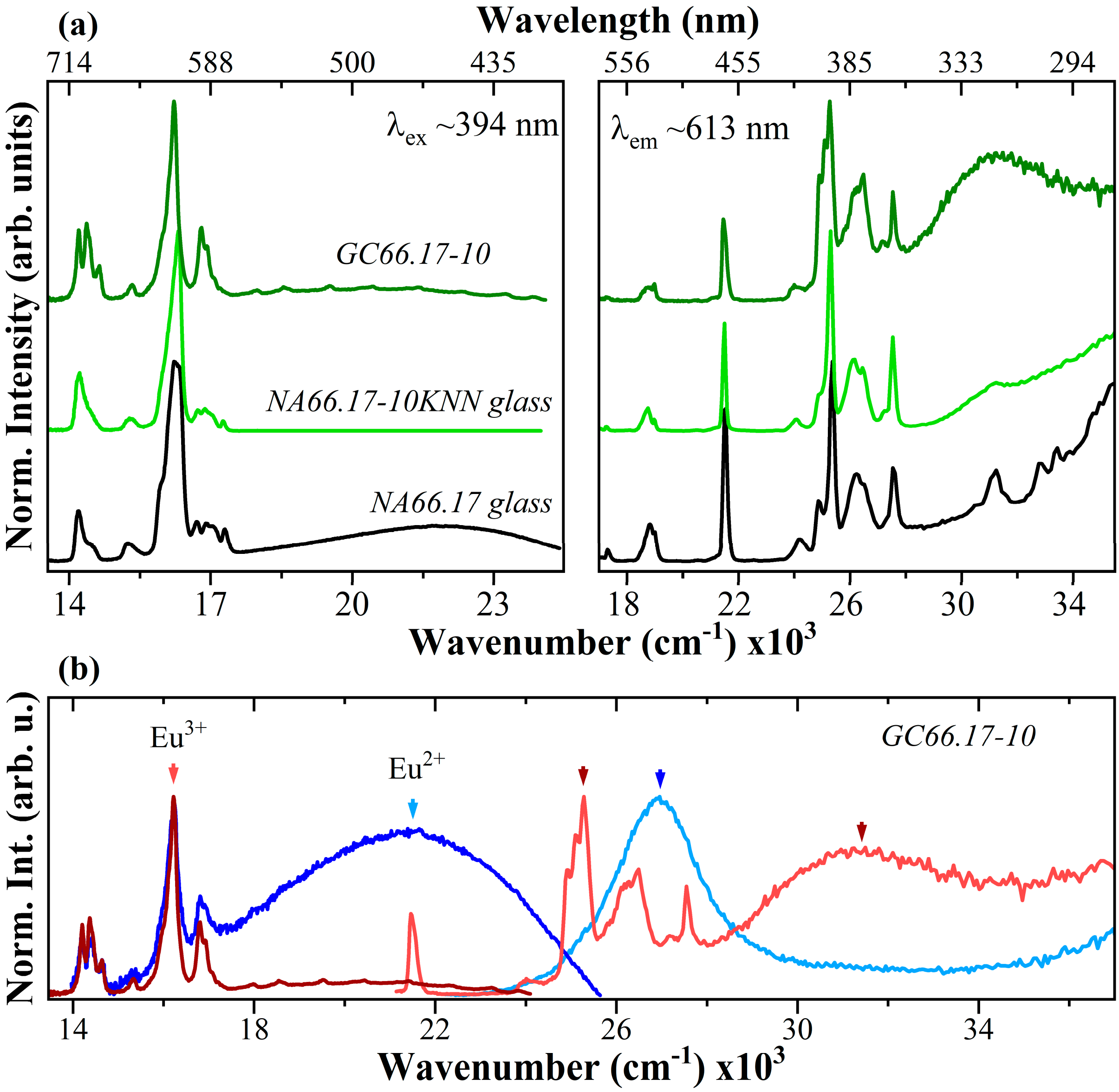

Figure S5. B: Photoluminescence emission (left panels) and excitation (right panels) spectra of the Eu-doped NA66.17 pristine glass (black lines), the glass NA66.17 with 10 mol% KNN (light green line) and the corresponding GC (GC66.17-10, olive green line). In the lower panel the excitation and emission spectra collected at the respective maxima (vertical arrows); the broad contributions associated with Eu^{2+} fluorescence in GC66.17-10 is visible.

Figure S6 – Energy Transfer

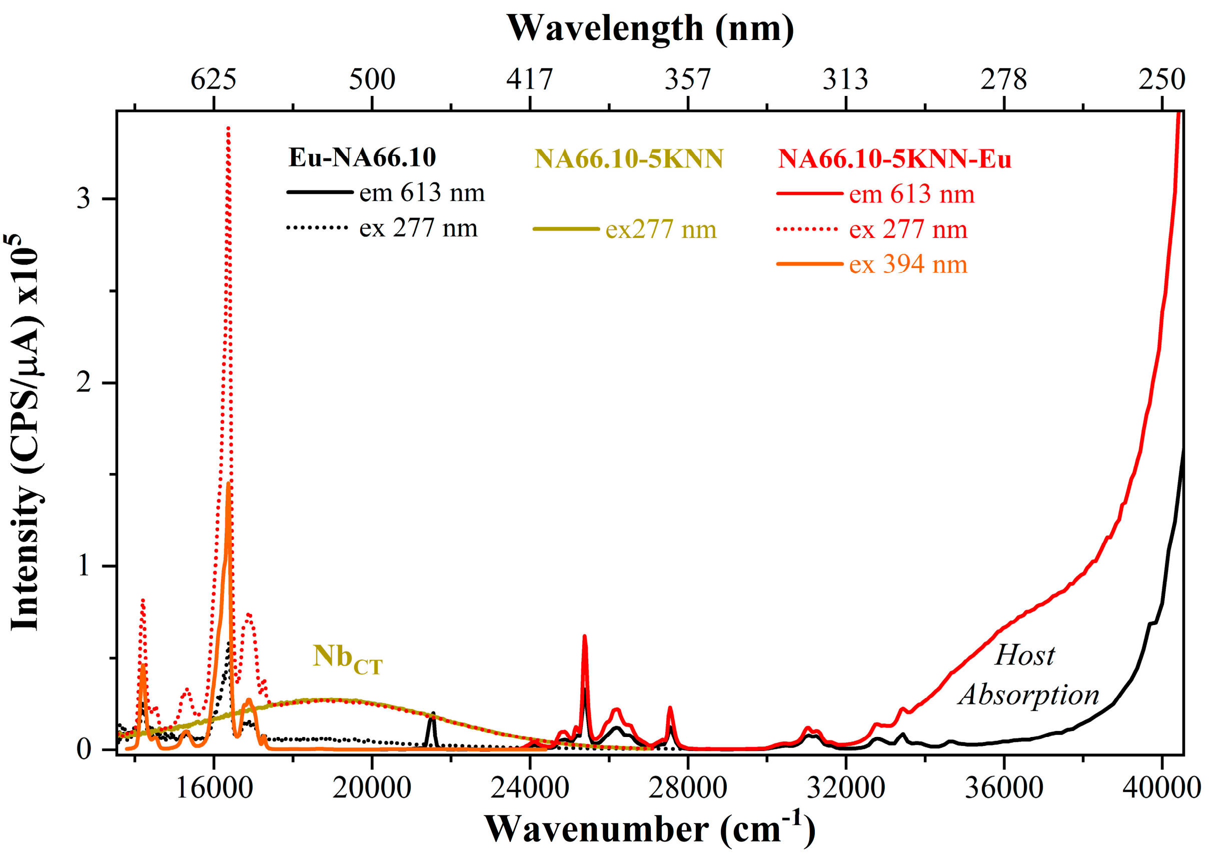

Figure S6. Room temperature photoluminescence emission and excitation spectra for the Eu-doped pristine glass NA66.10, the NA66.10-5KNN glass, and the same glass composition containing both Eu and 5 mol% KNN. Under an excitation of 277 nm, corresponding to the host absorption, the emission intensity of the Eu^{3+} transitions increases, along with the broad band related to the $O^{2-} \rightarrow Nb^{5+}$ charge transfer (Nb_{CT}). The intensity of the Eu^{3+} lines is much lower if Nb_2O_5 is not present in the glass matrix, as shown by the dotted black curve (Eu-NA66.10 under 277 nm excitation). These results show that there is the energy transfer from the host [NbO_6] to the Eu^{3+} ions.

APPENDIX:

Judd-Ofelt analysis for Eu^{3+} emission

In case of Eu^{3+} ion, it is possible to determine Judd-Ofelt parameters (Ω_λ : λ = 2, 4, 6) **[1,2]** from the analysis of emission spectra because the doubley reduced matrix elements corresponding the $^5D_0$-^7F_J transitions of Eu^{3+} ion, $||U^2||$ for J = 2, $||U^4||$ for J = 4, and $||U^6||$ for J = 6, are the only non-zero matrix elements.**[R3]** The radiative transition probability A_{ed} is given by **[3,4]**

$$A_{ed} = \frac{64\pi^4 e^2 \nu^3}{3h(2J'+1)}\left[\frac{n(n^2+2)^2}{9}\right]\sum_{\lambda}\Omega_\lambda\langle\, ^5D_{J'}\|U^\lambda\|\, ^7F_\lambda\rangle^2,$$

where $n(n^2+2)^2/9$ is the Lorentz local field correction, n being the refractive index of the medium, ν being the wavenumber of emission peak for $^5D_{J'}$-^7F_J transition, and h being planch constant. In the analysis, the intensity of allowed magnetic dipole $^5D_0$-$^7F_1$ emission transition, given by **[3,4]**

$$A_{md} = \frac{64\pi^4 \nu^3}{3h(2J'+1)} n^3 S_{md}$$

was taken as a reference, where S_{md} is the magnetic dipole line strength of $^5D_0$-$^7F_1$ transition and independent of host matrix. Thus, Ω_λ can be calculated from the ratio of the intensity of $^5D_0$-^7F_J (J = 2, 4, 6) transition, $\int I_{5D0-7FJ}dv$ dv, to the intensity of the $^5D_0$-$^7F_1$ transition, $\int I_{5D0-7F1}dv$,**[5,6]**

$$\frac{\int I_{5D0-7FJ}dv}{\int I_{5D0-7F1}dv} = \frac{e^2}{S_{mc}}\left(\frac{v_J}{v_1}\right)^3 \frac{n(n^2+2)^2}{9n^3}\Omega_J\langle\|U^J\|\rangle^2$$

S_{md} of $^5D_0$-$^7F_1$ was obtained from the data of A.X.Axes, Jr et al.**[7,8]**

Total radiation probability for $^5D_0$ level is given by

$$A_{Total} = A_{md}(\,^5D_0 \rightarrow \,^7F_1) + \sum_{J=2,4,6} A_{ed}(\,^5D_0 \rightarrow \,^7F_J),$$

and theoretical $^5D_0$ lifetime $\tau_r = 1/A_{Total}$ was calculated from the Ω_J parameters according to the above-mentioned method, which is compared with the experimental one to know luminescence efficiency η of $^5D_0$ level, which is shown in the main text.

References

1. Judd, B.R. Optical absorption intensities of rare-earth ions. *Phys. Rev.* **1962**, *127*, 750–761.
2. Ofelt, G.S. Intensities of crystal spectra of rare-earth ions. *J. Chem. Phys.* **1962**, *37*, 511–520.
3. Babu, P.; Jayasankar, C.K. Optical spectroscopy of Eu^{3+} ions in lithium borate and lithium fluoroborate glasses. *Physica B* **2000**, *279*, 262–281.
4. Walsh, B.M. Judd-Ofelt theory: Principles and practices. In *Advances in Spectroscopy for Lasers and Sensing*; Di Bartolo, B., Forte, O., Eds.; Springer: Dordrecht, The Netherlands, 2006. https://doi.org/10.1007/1-4020-4789-4_21.
5. Ebendorff-Heidepriem, H.; Ehrt, D. Spectroscopic properties of Eu^{3+} and Tb^{3+} ions for local structure investigations of fluoride phosphate and phosphate glasses. *J. Non-Cryst. Solids* **1996**, *208*, 205–216.
6. Maheswari, D.U.; Kumar, J.S.; Moorthy, L.R.; Jang, K.; Jayasimhadri, M. Emission properties of Eu^{3+} ions in alkali tellurofluorophosphate glasses. *Physica B* **2008**, *403*, 1690–1694.
7. Axe, J.D., Jr. Radiative transition probability within 4fn configurations: The fluorescence spectrum of Europium ethlysulfate. *J. Chem. Phys.* **1963**, *39*, 1154–1160.
8. Ikeshita, R.; Hayakawa, T.; Isogai, M.; Iwamoto, Y.; Duclère, J.-R.; Rémondière, F.; Thomas, P.; Lecomte, A. Novel method to control initial crystallization of Eu^{3+} doped ZrO_2 nanophosphors derived from a sol-gel route based on HNO_3 and their site-selective photoluminescence. *J. Ceram. Soc. Japan* **2018**, *126*, 551–556.
